# Renal hypouricemia complicated with kidney stone: a case report

**DOI:** 10.3389/fmed.2024.1218232

**Published:** 2024-02-07

**Authors:** Yuhao Yang, Xingyu Mu, Zengxiang Wu, Zhenmei An, Shuangqing Li

**Affiliations:** ^1^General Practice Ward/International Medical Center Ward, General Practice Medical Center, West China Hospital, Sichuan University, Chengdu, Sichuan, China; ^2^Department of Endocrinology and Metabolism, West China Hospital, Sichuan University, Chengdu, China

**Keywords:** renal hypouricemia, SLC22A12, mutation, asymptomatic kidney stones, next-generation sequencing

## Abstract

Renal hypouricemia (RHUC) is a rare autosomal recessive disorder characterized by impaired renal tubular uric acid reabsorption and abnormally high uric acid clearance, which may be manifested by reduced serum uric acid (SUA) levels and elevated fractional excretion of uric acid (FE-UA >10%). Most RHUC patients are often asymptomatic or have accidentally decreased SUA levels during health examinations, while others develop kidney stones and exercise-induced acute kidney injury (EIAKI). We now report a case of RHUC complicated with an asymptomatic kidney stone, and we identified a heterozygous mutation of c.269G > A (p.R90H) and a novel heterozygous mutation of c.674C > G (p.T225R) in the *SLC22A12* gene in the patient through whole exon gene detection (NGS method). This case offers valuable insights into the mechanisms, clinical management, and prognosis of RHUC and its associated complications.

## Introduction

**Figure 1:**
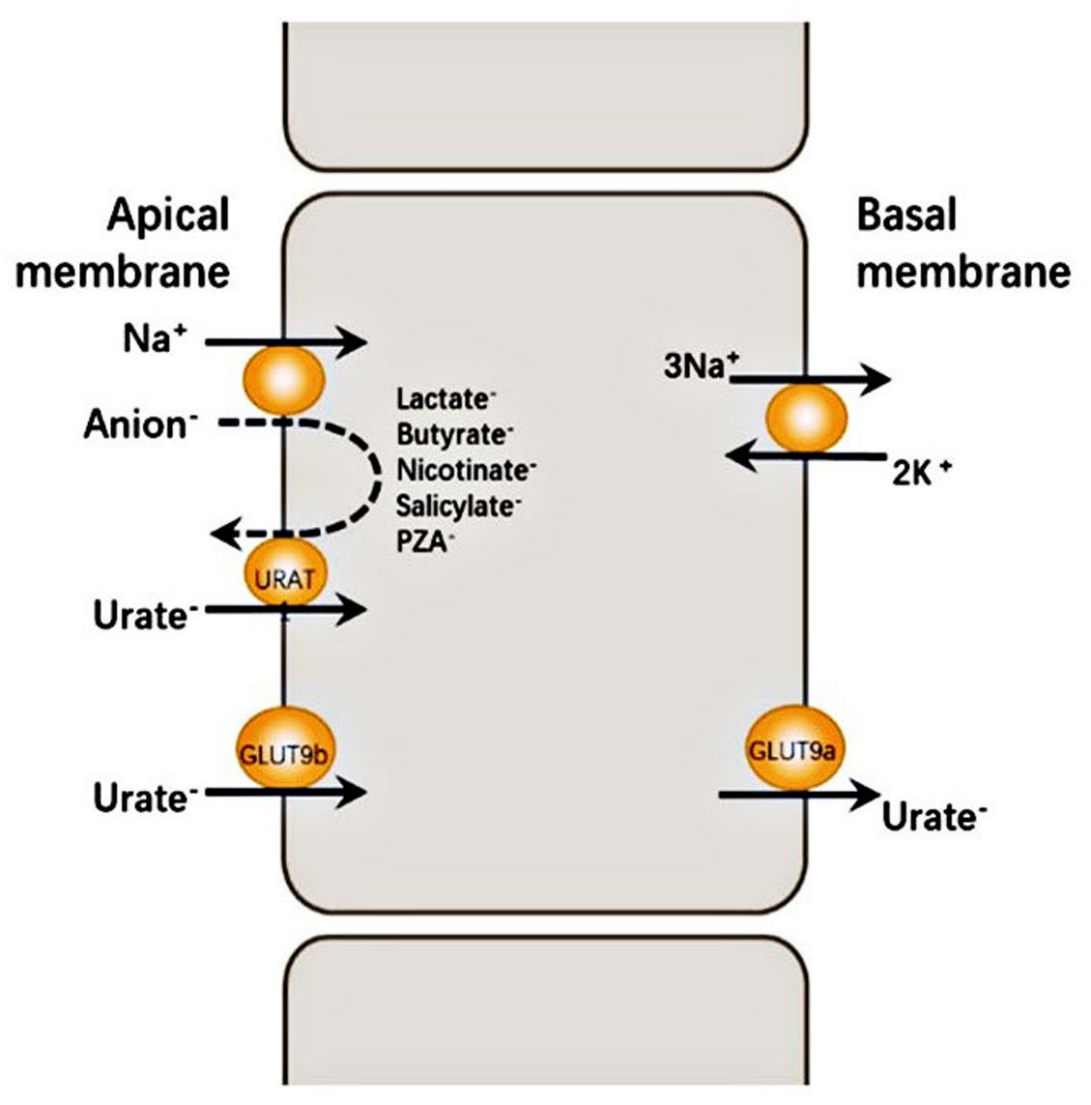
URAT1 reabsorbs urate from the glomerular ultrafiltrate by exchanging luminal urate with monovalent intracellular anions such as PZA^**−**^ and lactate^**−**^. The intracellular concentration of these anions is determined largely by Na^+^-dependent absorption from the same glomerular ultrafiltrate. GLUT9 is the major urate exit mechanism at the basolateral membrane of the proximal tubule, allowing for urate reabsorption into the blood. Human GLUT9 has two splice variants with distinct N-terminal isoforms: GLUT9a is expressed at the basal membrane of proximal tubule cells with GLUT9b at the apical membrane of proximal tubule cells.

Hypouricemia is defined as a serum urate concentration of <119 μmoL/L (2 mg/dL). This condition results from various factors, including decreased uric acid production, defective renal tubular reabsorption due to inherited or acquired disorders, or uric acid oxidation triggered by uricase treatment (1). Specifically, renal hypouricemia is a rare hereditary disease characterized by impaired uric acid transport, insufficient reabsorption, and accelerated secretion. Renal hypouricemia (RHUC) is now classified into two groups: RHUC1 [OMIM #220150], which results from a mutation in the *SLC22A12* [OMIM *607096], encoding for renal urate transporter 1 (URAT1), and RHUC2 [OMIM #612076], which results from a mutation in the *SLC2A9* [OMIM *606142], encoding for glucose transporter (GLUT9) (2). URAT1 and GLUT9 are expressed in the apical and basal membranes of the renal cortical proximal tubule, respectively. These two proteins are important transporters, facilitating renal uric acid reabsorption and upholding uric acid homeostasis. The mechanism for urate secretion by the renal cortical proximal tubule is shown in Figure 1 (3). In this study, we report a case of RHUC with an asymptomatic kidney stone. We identified the patient as having a heterozygous mutation of c.269G > A (p.R90H) and a novel heterozygous mutation of c.674C > G (p.T225R) in the *SLC22A12* gene through whole exon gene detection (NGS method) for the first time to our knowledge.

## Case report

**Figure 2:**
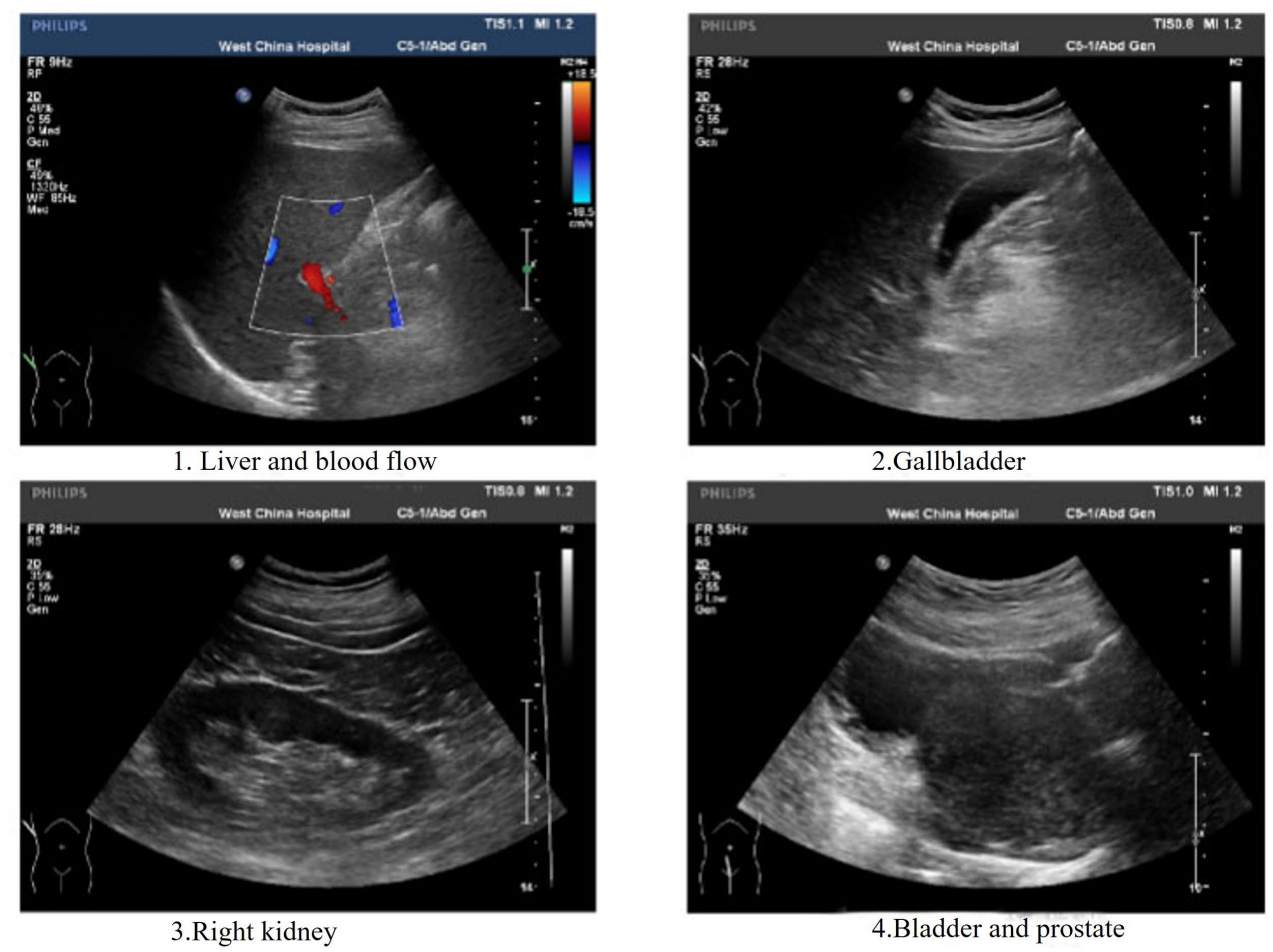
As observed in the color ultrasound images: Picture 1 shows a normal liver morphology, characterized by homogeneous parenchymal echogenicity and the absence of occupying lesions. In Picture 2, the gallbladder appears normal in size, with several slightly echogenic attachments on the wall, the largest measuring approximately 0.5 cm. No abnormal echoes are detected within the cystic cavity. Picture 3 reveals a strong echogenic mass of about 0.5 cm in the lower pole of the right kidney, with no distinct hypoechoic areas in the collecting system. Moving to Picture 4, the anteroposterior diameter of the prostate is approximately 5.3 cm, protruding into the bladder by about 1.8 cm, and displaying heterogeneous parenchymal echogenicity.

**Figure 3:**
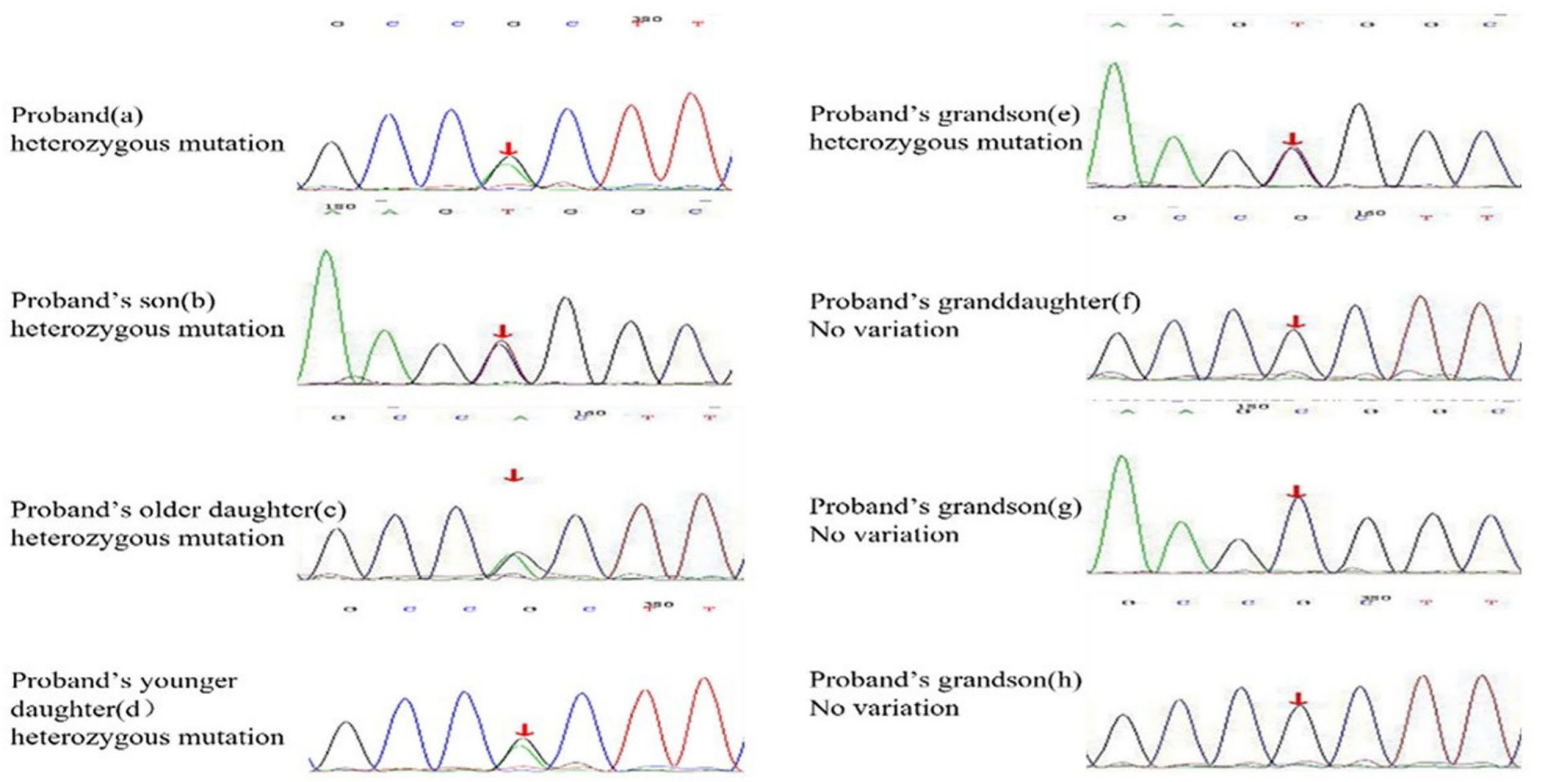
Heterozygous mutation at nucleotide 674 from cytosine C to guanine G(c.674C > G) is found in proband, proband’s son, proband’s two daughters, and proband’s grandson(e). The rest of the proband’s families have no variation at the SLC22A12 gene.

**Figure 4:**
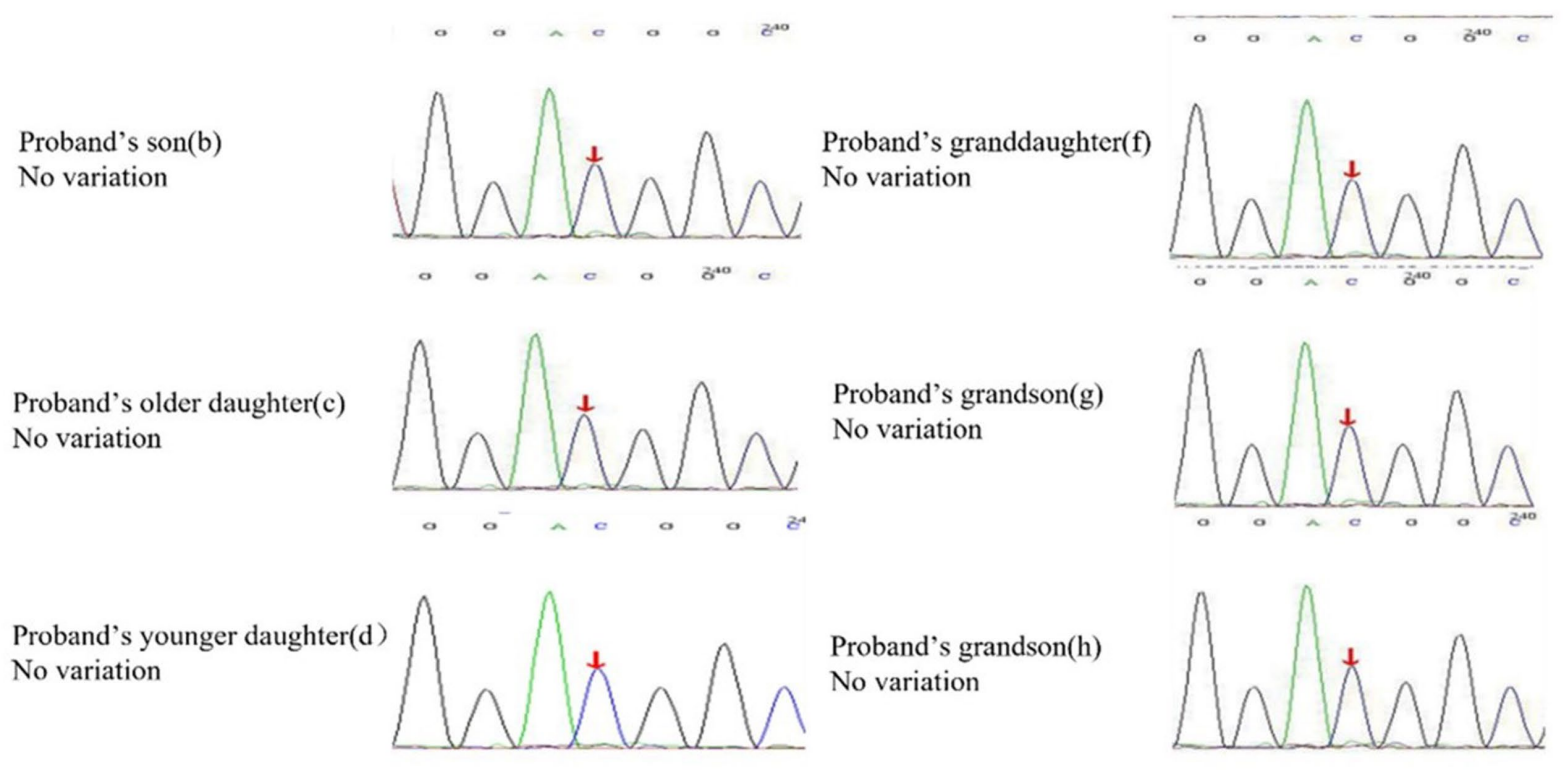
Heterozygous mutation at nucleotide 674 from cytosine C to guanine G(c.674C > G) is found in proband, but all of his offspring have no variation in the SLC22A12 gene.

**Figure 5:**
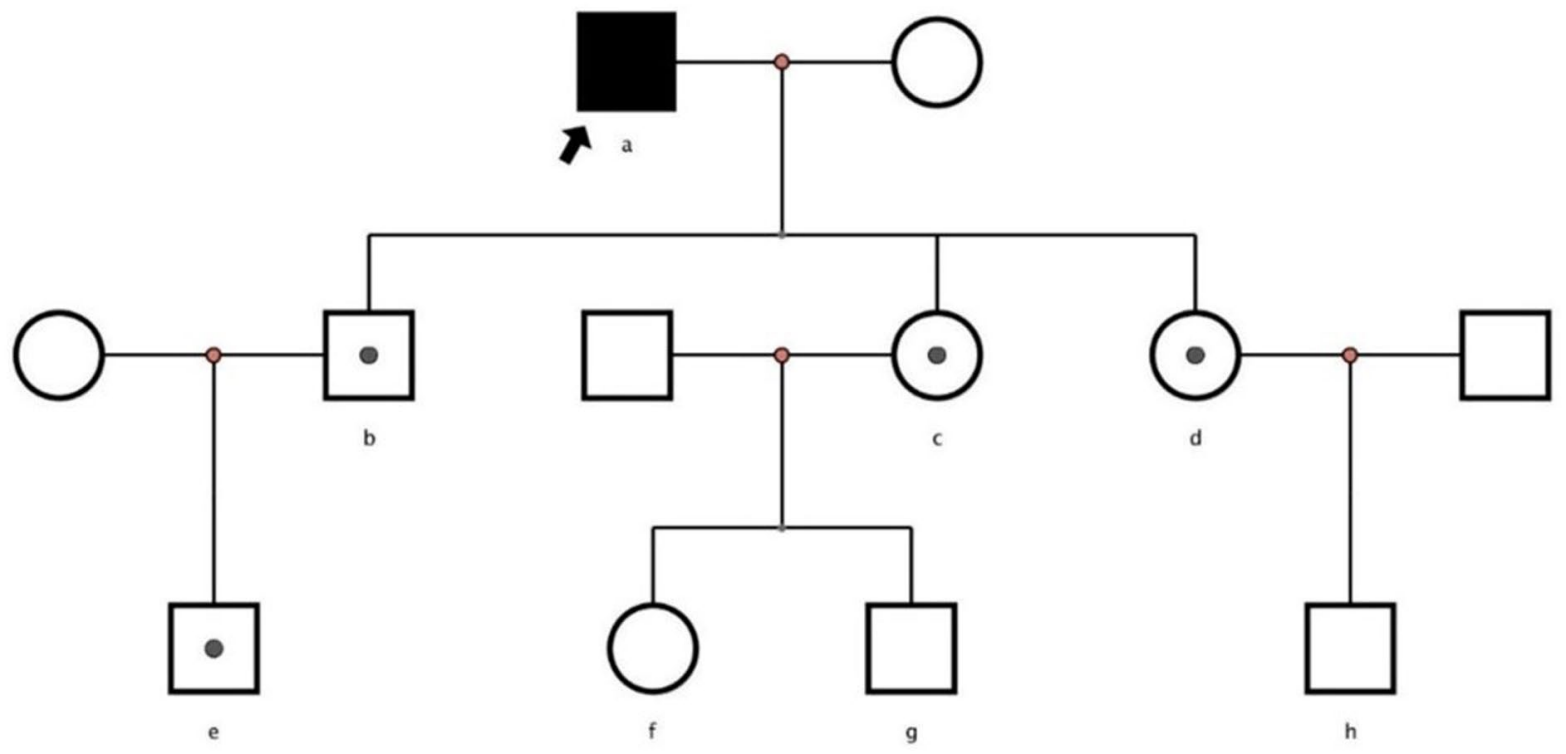
Pedigrees of families with renal hypouricemia and SLC2A12 mutations. Solid symbols denote affected family members, white symbols square healthy members and unexamined family members, and white symbols with a solid circle inside denote heterozygous.

A 62-year-old retired man presented to the Outpatient Department of Endocrinology and Metabolism of our hospital on 18 July 2022, because of “low serum uric acid and kidney stones found in a physical examination for 4 years.” Four years before admission, a physical examination was performed in the local hospital, and “low serum uric acid, kidney stone” was found. The patient had no symptoms of lower back pain and distension, abdominal pain, or hematuria and had not taken medications to reduce uric acid and promote uric acid excretion. Two years before admission, serum uric acid was reexamined at 17 μmol/L (normal range: 134–415 μ mol/L) in the local hospital. On ultrasound examination of the urinary system, a hyperechoic lesion with a size of approximately 0.5 × 0.4 cm was observed at the lower pole of the right kidney. The color ultrasound diagnosed “a right kidney stone.” His current visit to our hospital was prompted by the desire for a comprehensive diagnosis and treatment plan. Other medical histories: 2 years before admission, during the health examination, the patient was found to have a venous fasting blood glucose of 7.69 mmol/L (normal range 3.9–6.1 mmol/L) and no symptoms of polydipsia, polyphagia, polyuria, or weight loss. He was diagnosed with “Type 2 diabetes” in the local hospital and had taken “metformin” for half a year. After that, he stopped taking the medicine on his own and did not regularly monitor his blood glucose. Outpatient physical examination: T: 36.3°C, P: 83 times/min, R: 16 times/min, BP:120/85 mmHg; height: 173 cm, weight: 85 kg, BMI: 28.40 kg/m^2^; cardiac and pulmonary and abdominal physical examination showed no obvious abnormality. Laboratory tests in our hospital showed kidney function impairment (serum creatinine 59 μmol/L), a low SUA level (serum uric acid 34 μmol/L), and a high FE-UA level (16%). Details of other laboratory tests are shown in Table 1. Color ultrasound of the urinary system: a hyperechoic lesion approximately 0.5 cm in size was found in the middle and lower calyces of the right kidney (Figure 2). Preliminary diagnosis: (1) renal hypouricemia; (2) type 2 diabetes; and (3) right kidney stone. The cause of hypouricemia was not clear. On 22 July 2022, the peripheral blood of the patient and his family members was collected, and the specimens were sent to the gene examination laboratory for whole exon gene detection (NGS method). On 22 August 2022, highly suspicious mutations of the *SLC22A12* gene related to RHUC were found in the gene test report. There were two heterozygous mutations in the *SLC22A12* gene of this patient: the heterozygous mutation at nucleotide 269 changed from guanine G to adenine A (c.269G > A), which resulted in an amino acid change of p.R90H. Three children (b, c, and d) and one grandson (e) of this patient also had a heterozygous mutation at nucleotide 269 (Figure 3). In accordance with the guidelines of the American College of Medical Genetics and Genomics (ACMG), this mutation was preliminarily classified as a pathogenic mutation. The heterozygous mutation at nucleotide 674 from cytosine C to guanine G(c.674C > G) resulted in an amino acid change of p.T225R. According to ACMG guidelines, this mutation was preliminary classified as having unknown clinical significance, and all of his family members had no heterozygous mutation at this site (Figure 4). Because the patient’s parents had passed away, no samples of them had been collected, and the source of the genetic mutation had not been verified. Combined with the patient’s medical history, laboratory test results, and genetic test results, RHUC1 was diagnosed. The pedigree of this whole family c.269G > A mutation in the *SLC2A12* was depicted (Figure 5).

**Table 1:** Laboratory tests at hospital admission.

Parameter	Results	Normal value	Parameter	Results	Normal value
RBC	4.75 × 10^12^/L	4.3–5.8 × 10^12^/L	SUA	34 μmol/L	240–490 μmol/L
HB	143 g/L	130–175 g/L	TG	0.88 mmol/L	0.29–1.83 mmol/L
PLT	234 × 10^9^/L	100–300 × 10^9^/L	CHOL	4.75 mmol/L	2.80–5.70 mmol/L
WBC	4.27 × 10^9^/L	3.5–9.5 × 10^9^/L	HDL-C	1.31 mmol/L	>0.90 mmol/L
TBIL	21.0 μmol/L	5.0–28.0 μmol/L	LDL-C	3.14 mmol/L	<4.0 mmol/L
DBIL	4.9 μmol/L	<8.8 μmol/L	NA	140.1 mmol/L	137.0–147.0 mmol/L
IBIL	16.1 μmol/L	<20 μmol/L	K	4.37 mmol/L	3.50–5.30 mmol/L
ALT	19 IU/L	<50 IU/L	GLU0	7.21 mmol/L	3.9–5.9 mmol/L
AST	20 IU/L	<40 IU/L	GLU120	15.31 mmol/L	3.3–7.8 mmol/L
ALP	42 IU/L	51–160 IU/L	INS0	14.3 μU/mL	1.5–15.0 μU/ml
GGT	19 IU/L	<60 IU/L	INS120	122.0 μU/mL	3.0–60.0 μU/ml
TP	72.0 g/L	65.0–85.0 g/L	HbA1c	6.4%	4.5–6.1%
ALB	49.9 g/L	40.0–55.0 g/L	TSH	2.250 mIU/L	0.27–4.2 mIU/L
GLU	7.23 mmol/L	3.90–5.90 mmol/L	FT4	17.70 pmol/L	12.0–22.0 pmol/L
UREA	4.3 mmol/L	3.6–9.5 mmol/L	PTH	3.42 pmol/L	1.60–6.90 pmol/L
CREA	59 μmol/L	68–108 μmol/L	24HU	3.30 L/24 h	**——**
eGFR	103.14 mL/min/1.73m^2^	**——**	24HUA	5.87 mmol/24 h	2.4–5.9 mmol/24 h

## Discussion

RHUC is characterized by impaired renal tubular uric acid reabsorption and abnormally high uric acid clearance, resulting from pathogenic mutations in either *SLC22A12*, which encodes URAT1 (RHUC1), or *SLC2A9*, which encodes GLUT9 (RHUC2). Loss-of-function mutations in *SLC22A12* constitute the primary cause of RHUC, accounting for over 90% of cases (4). Notably, population-specific common variants have been identified in hypouricemia among Japanese, Korean, and Roma patients. In the Japanese population, there are two prevalent RHUC1 allelic variants: c.774G > A (p.W258X) with a frequency of 2.30–2.37%, and c.269G > A (p.R90H) with a frequency of 0.40%. Hence, the total frequency of URAT1 allelic variants in Japan is approximately 3%. The prevalence of allelic variants in the Korean population is similar to that in the Japanese population (4). In a study of the European Roma population, Stiburkova et al. found that the most common RHUC1 allelic variants are c.1245_1253del (p.L415_G417del) with a frequency of 1.92% and c.1400C > T (p.T467M) with a frequency of 5.56%. Interestingly, the prevalence of RHUC1 allelic variants in this population is even higher than in Japan or Korea (5). In China, there have been reports of 10 patients with mutation alleles (heterozygous and/or compound heterozygous and/or homozygous) in the *SLC22A12* gene and 16 patients with heterozygous and/or compound heterozygous and/or homozygous mutant alleles in the *SLC2A9* gene (including those in the present study, Table 1). Our findings indicate that the mutant alleles of c.269G > A (p.R90H) are prevalent in the *SLC22A12* gene, accounting for 70% (7/10, Table 1), and the mutant alleles of c.857G > A (p.W286X) are prevalent in the *SLC2A9* gene, constituting 43.75% (7/16, Table 1). Therefore, we hypothesize that the p.R90H and p.W286X mutations may play crucial roles as major contributors to Chinese RHUC1 and RHUC2 patients, respectively. However, a definitive conclusion cannot be drawn at this point due to the limited number of known mutations in China and the influence of familial hereditary factors.

The laboratory test indexes for RHUC patients are typically SUA < 2.0 mg/dL (120 moL/L) and FE-UA > 10% (6). In comparison to RHUC1, RHUC2 exhibits a broader clinical variability: in individuals with compound heterozygous and/or homozygous RHUC2, SUA levels are significantly lower (often ranging from 0.1 to 0.2 mg/dL, and sometimes approaching 0 mg/dL), with FE-UA markedly higher (frequently >150%). This can be explained by the complete halt of the resorption mechanism of the basal lateral membrane in individuals with homozygous or compound heterozygous mutations in GLUT9/*SLC2A9*. The reduced uric acid resorption in the apical membrane leads to a significant increase in uric acid excretion (7). Conversely, heterozygous RHUC2 family members maintain normal uric acid levels. It is noteworthy that heterozygous GLUT9 mutations lead to hypouricemia haploinsufficiency rather than exhibiting dominant-negative effects (8). Furthermore, individuals with *SLC2A9* mutations, especially those who are homozygous, appear to be more susceptible to EIAKI and kidney stones (9). The typical symptoms of EIAKI are acute abdominal pain, nausea, and impaired renal function within 6–12 h after exercise (10). The mechanism behind how RHUC leads to EIAKI is not yet fully understood. Some researchers have suggested potential explanations in the past. One theory is that the buildup of uric acid crystals in the renal tubules causes acute kidney injury. Another theory is that during intense exercise, a shortage of uric acid as a reducing agent leads to a surge in ROS, causing renal vasoconstriction and acute ischemic kidney injury (11). Miyamoto D et al. recently conducted a semi-ischemic forearm exercise stress test, and their findings suggest that the hypotheses mentioned earlier are still controversial. Their analysis indicates an association with hypoxanthine (HX) in the pathogenesis of EIAKI. HX serves as a substrate in the salvage pathway. The observed reduction in blood HX after ischemic exercise stress in renal hypouricemia subjects, compared to healthy participants and individuals with hereditary xanthinuria, indicates their inability to resynthesize ATP through IMP. This implies their heightened sensitivity to stress-induced hypoxia. While this new possible mechanism is still being confirmed, it provides a new research direction for clarifying EIAKI in RHUC patients (12).

Analyzing mutations through bioinformatics might help explain the related causes of RHUC (13). Previous experiments showed that changing one amino acid to p.R90H significantly lowers URAT1’s ability to transport uric acid, but it does not affect how it is produced or where it is located in the cell membrane (14). Zhou et al. did URAT1 protein domain analysis. They did molecular modeling, predicting that when the mutation at nucleotide 269 changes from guanine G to adenine A (c.269G > A), resulting in an amino acid change p.R90H, the mutation would have a certain effect on protein structure. In the wild model, Arg90 forms strong hydrogen bonds with Cys88 and Gln93 to maintain the conformational stability of the intracellular domain. In mutant models, the formation of hydrogen bonds between His90 and Gln93 is weak, which may reduce the stability of this region and may weaken the coupling between URAT1 and other proteins (such as PDZK1), thereby affecting the transport of uric acid (15). Therefore, this molecular modeling suggests that the structural instability of the protein caused by the gene mutation may be one of the primary mechanisms underlying RHUC.

In patients with RHUC and kidney stones (including the patient mentioned in this article), surgical intervention is recommended for symptomatic cases, urinary obstruction, infection recurrences, or when stone size increases, particularly if stones exceed 10 mm or are accompanied by renal anomalies. For asymptomatic patients, surgical intervention is typically not required, but surveillance imaging every 1 or 2 years with kidney ultrasonography or CT can identify patients at risk for symptomatic passage (16). Lifestyle modifications are crucial, including elevating daily fluid intake to 2.5–3 liters for optimal diuresis, minimizing the intake of phosphate-rich carbonated drinks, and adjusting the diet to favor fruits and vegetables while reducing non-dairy animal proteins. Regularly maintaining a urinary pH between 6.0 and 7.5 is essential for stone management. Potassium citrate is advised for urinary alkalization, effectively inhibiting stone growth and recurrence and facilitating the dissolution of existing stones (17). The comprehensive approach to managing RHUC and its complications, including kidney stones, becomes even more complex when considering other coexisting conditions such as diabetes. According to Spatola et al., insulin resistance is negatively associated with renal ammonium synthesis and urine pH, which increases the risk of developing uric acid stones, calcium oxalate stones, or a combination of both (18). Moreover, the uricosuric effect of glycosuria, a potential biological mechanism, could lead to extreme hypouricemia (19). Therefore, it is necessary for RHUC patients with diabetes to strictly control their blood glucose (Table 2).

**Table 2:** SLC22A12 and SLC2A9 mutation patients and their families in China.

Patient	Gender (M^a^/F^b^)	Age	FEUA(%)	Clinical symptoms	Type	SLC22A12 variants nucleotide amino acid	SLC2A9 variants nucleotide amino acid
1)	M	27	46.6	EIAKI	Compound Heterozygous	c.626C > T/ c.1372G > A (p.A209V/p.E458K)	
2)	M	23	-	EIAKI	Heterozygous	c.151delG(p.A51fsX)	
3)	M	62	16	Kidney Stones	Compound Heterozygous	c.269G > A/c.674C > G (p.R90H/(p.T225R)	
4)	M(3’son)	-	-	No	Heterozygous	c.269G > A(p.R90H)	
5)	F(3’daughter)	-	-	No	Heterozygous	c.269G > A(p.R90H)	
6)	F(3’daughter)	-	-	No	Heterozygous	c.269G > A(p.R90H)	
7)	M(3’grandson)	-	-	No	Heterozygous	c.269G > A(p.R90H)	
8)	F	27	50	No		c.269G > A/ c.1289_1290insGG (p.R90H/p.M430fsX466)	
9)	M(8’father)	-	-	No	Compound Heterozygous	c.1289_1290insGG(p.M430fsX466)	
10)	F(8’mother)	-	-	No	Heterozygous	c.269G > A(p.R90H)	
11)	M	35	>150	EIAKI	Homozygous		c.857G > A(W286X)
12)	M(11’brother)	-	-	No	Heterozygous		c.857G > A(W286X)
13)	M(11’father)	-	-	No	Heterozygous		c.857G > A(W286X)
14)	M(11’son)	-	-	No	Heterozygous		c.857G > A(W286X)
15)	F(11’mother)	-	-	No	Heterozygous		c.857G > A(W286X)
16)	F(11’Neice)	-	-	No	Heterozygous		c.857G > A(W286X)
17)	M	32	74	EIAKI	Compound Heterozygous		c.68C > T/c.857C > T(W23X/W286X)
18)	M	11	139.4	EIAKI, PRES	Homozygous		c.1215 + 1G > A(#)
19)	F(18’mother)	-	-	-	Heterozygous		c.1215 + 1G > A(#)
20)	M(18’brother)	-	-	-	Heterozygous		c.1215 + 1G > A(#)
21)	F	12	>150	EIAKI	Homozygous		g.68G > A(p.Trp23Stop)
22)	M(21’brother)	8	24.72	No	Heterozygous		g.68G > A(p.Trp23Stop)
23)	M(21’father)	-	-	No	Heterozygous		g.68G > A(#)
24)	F(21’mother)	-	-	No	Heterozygous		g.68G > A(#)
25)	M	8	12.7	EIAKI, Kidney Stones	Heterozygous		c.595-2_595-linsC(#)
26)	M(25’brother)	-	5.5	Kidney Stones	Heterozygous		c.595-2_595-linsC(#)

M^a^ refers to male, and F^b^ refers to female.

In summary, by using the NGS method of this patient and his family members, we found out that this family has a heterozygous mutation at c.269G > A of *SLC22A12*, which is a pathogenic mutation of RHUC1, and the patient has a novel heterozygous mutation at c.674C > G of *SLC22A12*, the clinical meaning of which is currently uncertain. We have conducted a thorough review of the literature to determine the current prevalence of the two major mutant alleles of RHUC in typical regions. Additionally, we have analyzed Chinese patients with mutations in *SLC22A12* and *SLC2A9* and identified the most common mutation types in China. Our research discusses potential reasons for the differences in clinical symptoms and laboratory tests between the two types of RHUC and presents the clinical manifestations and possible mechanisms of EIAKI. We have also introduced a molecular model that illustrates the abnormal uric acid transport resulting from the p.R90H mutation, which induces a structural alteration in the protein. Finally, we have summarized the management, treatment, and lifestyle changes for RHUC patients with kidney stone formation. However, we have found that the current awareness of RHUC remains limited, likely due to the scarcity of identified patients and related studies worldwide. Therefore, further research is necessary to elucidate this disease’s pathogenesis, treatment options, and prognosis to establish a consensus.

## Data availability statement

The original contributions presented in the study are included in the article/supplementary material; further inquiries can be directed to the corresponding author.

## Ethics statement

Ethical review and approval was not required for the study on human participants in accordance with the local legislation and institutional requirements. Written informed consent from the patients/participants was not required to participate in this study in accordance with the national legislation and the institutional requirements. Written informed consent was obtained from the individual(s) for the publication of any potentially identifiable images or data included in this article.

## Author contributions

YY, XM, ZW, and ZA contributed to conception and design of the study. YY and XM complete related data collection. ZW complete the analysis of relevant data. YY wrote the first draft of the manuscript. XM, ZW, and ZA wrote sections of the manuscript. SL works as the corresponding author to ensure that the descriptions are accurate and agreed by all authors. All authors contributed to the article and approved the submitted version.
